# *Artemisia herba*-*alba* Essential Oil: Chemical Composition, Phytotoxic Activity and Environmental Safety

**DOI:** 10.3390/plants14020242

**Published:** 2025-01-16

**Authors:** Beáta Baranová, Daniela Gruľová, Flavio Polito, Vincent Sedlák, Mária Konečná, Marta Mydlárová Blaščáková, Ismail Amri, Vincenzo De Feo, Janka Poráčová

**Affiliations:** 1 Department of Ecology, Faculty of Humanities and Natural Sciences, University of Prešov, 17 Novembra 1, 08001 Prešov, Slovakia; beata.baranova@unipo.sk (B.B.); daniela.grulova@unipo.sk (D.G.); defeo@unisa.it (V.D.F.); 2 Department of Pharmacy, University of Salerno, Via Giovanni Paolo II. 132, Fisciano, 84084 Salerno, Italy; 3 Department of Biology, Faculty of Humanities and Natural Sciences, University of Prešov, 17 Novembra 1, 08001 Prešov, Slovakia; vincent.sedlak@unipo.sk (V.S.); maria.konecna@unipo.sk (M.K.); marta.blascakova@unipo.sk (M.M.B.); janka.poracova@unipo.sk (J.P.); 4 Laboratory of Biotechnology and Nuclear Technology, National Center for Nuclear Sciences and Technologies (CNSTN), Sidi Thabet 2020, Tunisia; amri_amri@live.fr

**Keywords:** *Artemisia herba*-*alba*, essential oil, composition, phytotoxicity, environmental safety

## Abstract

Weeds cause a decrease in the quantity and quality of agricultural production and economic damage to producers. The prolonged use of synthetic pesticides causes problems of environmental pollution, the possible alteration of agricultural products and problems for human health. For this reason, the scientific community’s search for products of natural origin, which are biodegradable, safe for human health and can act as valid alternatives to traditional herbicides, is growing. Essential oils can have useful implications in agriculture by acting as effective alternatives to chemical herbicides. In this work, the chemical composition of an EO from *Artemisia herba*-*alba* and its herbicidal properties were studied on two weeds (*Lolium multiflorum* and *Trifolium pratense*) and two crops (*Brassica napus* and *Hordeum vulgare*) and its environmental safety was also assessed using three model organisms: *Chaoborus* sp., *Tubifex tubifex* and *Eisenia foetida*. The principal component of the EO was camphor (26.02%), with α- and β-thujone (9.60 and 8.38%, respectively), 1,8-cineole (8.02%), piperitenone (5.29%) and camphene (4.95%) as the main components. The EO demonstrated variable phytotoxic effects with a dose-dependent manner, inhibiting both the germination and the radical elongation of the tested seeds, and was also found to be environmentally safe for the selected organisms. The results lay the foundation for considering this EO as a potential weed control agent.

## 1. Introduction

A serious problem that afflicts the world of agricultural production and with which growers must continuously fight is that of weeds. The growth of weed plants within cultivated territories intended for production for human consumption causes a decrease in the quantity and quality of agricultural production, disturbing the growth of desired products and therefore causing economic damage to producers [1]. To address this problem, a series of chemical and synthetic herbicides have been developed over the years. However, although they act effectively, their massive and prolonged use causes environmental pollution, both of soil and water, and the possible alteration of agricultural products, with a consequent potential risk to human health [2]. For this reason, there is a growing interest among the scientific community in the search for products of natural origin, which are biodegradable, safe for both the environment and human health and can act as valid alternatives to traditional herbicides [3]. Plants produce secondary metabolites—bioactive compounds not directly involved in primary growth and development processes, but with significant ecological roles. Among them, some influence the growth of other plants, generating positive or negative effects known as allelopathy. This phenomenon, mediated by chemicals released into the environment, contributes to the regulation of plant interactions and ecosystem dynamics [4]. This phenomenon may have useful implications in the agricultural field as existing processes in nature can be exploited to search for valid alternatives to chemical herbicides, thus avoiding their demonstrated toxicity [3]. Essential oils (EOs) are complex mixtures of molecules, mainly volatile, capable of exerting numerous biological activities. They are used by plants as a means of communication and interaction and can be released into the environment to influence the behavior of other plants [5,6]. Although EOs can exert significant biological effects, they are generally not considered toxic at the concentrations used for therapeutic, cosmetic or environmental purposes, and they have low persistence in the environment and do not accumulate in food chains [7]. However, it should not be overlooked that their characteristics such as the hydrophobicity and prolonged integrity of the vesicles can have a significant impact on the environment on organisms present therein. For this reason, it is useful to evaluate the possible ecotoxic effects that the application of an EO in nature could have [8,9].

*Artemisia herba*-*alba* Asso, also known as desert wormwood, is a resilient, perennial dwarf shrub belonging to the Asteraceae family. It grows in arid and semi-arid areas of Spain, North Africa and the Middle East [10]. It plays an important role in the traditional medicine of various cultures due to its numerous therapeutic properties: antidiabetic, antihypertensive, antioxidant, antifungal, antimalarial, antispasmodic and many others [11,12,13,14,15,16].

The available literature reports significant variability in the composition of the EO of *A. herba*-*alba*, depending on the geographical region, the harvest period or the specific plant organ used for the EO extraction [17,18]. In general, the EOs from different locations in North Africa such as Algeria and Tunisia were found to belong to the chemotype thujone, camphor and, more rarely, eucalyptol [19,20]. Among the various components, thujone is the one that raises the most concerns due to its harmfulness. The World Health Organization (WHO) [21] and the Scientific Committee on Food of the European Commission [22] conducted a review on the acute and chronic toxicity of thujone. The European Parliament and Council regulation [23] prohibited the use of chemically pure thujone directly added to foods, though it may be indirectly introduced through the use of thujone-containing plants. Thus, thujone is banned as a food additive in the USA and its presence in foods and beverages is regulated in several countries [22]. The content and biological effects of thujone have been investigated using plant materials from *Artemisia absinthium* L., *Salvia officinalis* L., *Tanacetum vulgare* L. and *Thuja occidentalis* L. [19,22,24]. Since the 1800s, thujone has been recognized as a potent neurotoxin. Risk assessment for thujone, a primary component in herbal formulations, involves substantial uncertainties and challenges [25]. α-Thujone exhibits neurotoxic effects in rats and has antinociceptive activity in mice, and cases of human poisoning have been linked to the ingestion of wormwood oil containing α-thujone [26,27,28,29]. This monoterpenoid occurs in many plants, including different *Artemesia* species. In *Artemisia absinthium* L. and herbal medicines, α-thujone acts as a fast-acting and quickly detoxified modulator of the GABA-gated chloride channel [30,31]. Wormwood extracts have been used for controlling gastrointestinal worms, with documented use dating back to ancient Egypt. *A. absinthium* and wormwood oil exhibit insecticidal properties, with α-thujone identified as one of the two most toxic monoterpenoids tested against larvae of the western corn rootworm [32,33]. In addition to neurotoxic effects, thujones may exhibit genotoxic and carcinogenic properties; however, they can also demonstrate antimutagenic, immunomodulatory and anticarcinogenic effects [34,35,36,37,38]. Growing public distrust of synthetic drugs and pesticides has increased the popularity of naturally occurring drugs and herbicides, even though these alternatives are not subjected to the same rigorous safety testing and toxicological evaluation [39,40,41].

In this work, the chemical composition of an EO from Tunisian *A. herba*-*alba* and its herbicidal properties were studied on two globally important weeds (*Lolium multiflorum* Lam., annual ryegrass and *Trifolium pratense* L., red clover) and two crops (*Brassica napus* L., rapeseed and *Hordeum vulgare* L., barley). Due to the significant presence of thujone in the EO, three model organisms (*Chaoborus* spp., *Tubifex tubifex* and *Eisenia foetida*), important indicators of water and soil health and representatives of aquatic, semi-aquatic and soil organisms, were exposed to the identical concentrations of EO applied in the phytotoxicity test. In the discussion section (Section 3), it is seen how the results of this work are partially in agreement with what is present in the literature regarding the chemical composition of the EO; in fact, compounds such as camphor and thujone are recognized among the main components in many EOs of *A. herba*-*alba*. The results are in strong agreement regarding the phytotoxic activity, although the seeds considered are different, the EO of *A. herba*-*alba* being recognized to have strong phytotoxic activity. The results provided information on the phytotoxic potential of the EO and its possible ecotoxic impact.

## 2. Results

### 2.1. Chemical Composition

The EO analysis highlighted the presence of 81 compounds, accounting for 97.98% of the total. The main class was that of oxygenated monoterpenes (79.35%). The compound present in the highest quantity was camphor (26.02%), the other main components being α- and β-thujone (9.60 and 8.38%, respectively), 1,8-cineole (8.02%), piperitenone (5.29%) and camphene (4.95%) (Table 1).

### 2.2. Phytotoxic Activity

**Figure 1:**
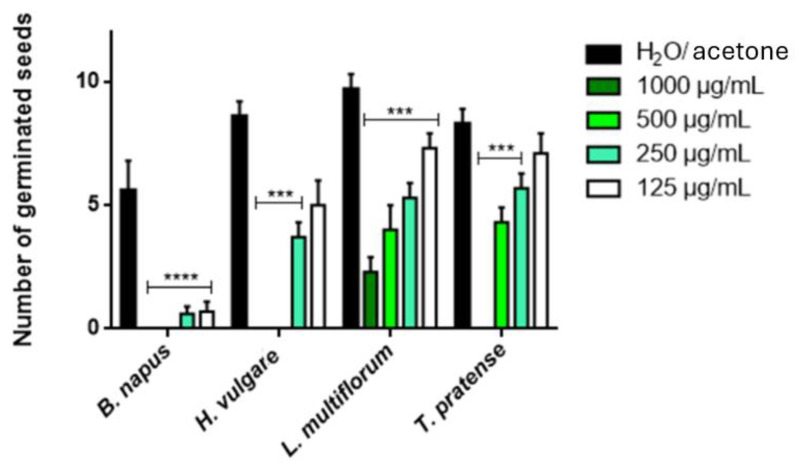
Phytotoxic activity of *A. herba*-*alba* EO against the germination of *B. napus*, *H. vulgare*, *L. multiflorum* and *T. pratense.* The results are the mean of three experiments ± SD. *** *p* < 0.001; **** *p* < 0.00001 compared with the control (ANOVA followed by Dunnet’s multiple comparison test).

**Figure 2:**
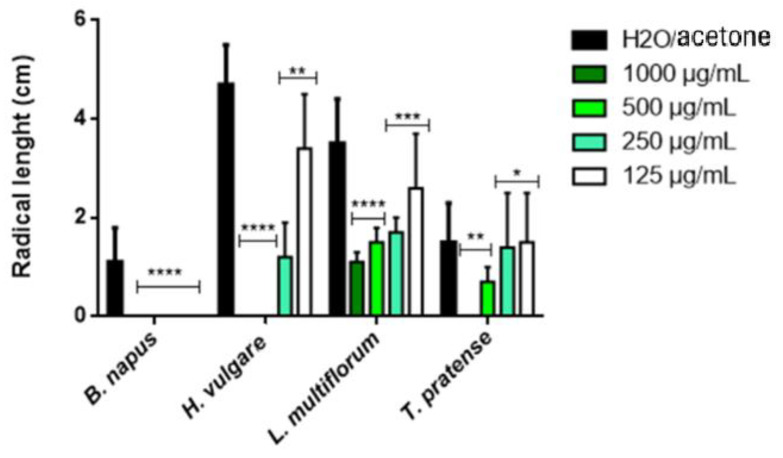
Phytotoxic activity of *A. herba*-*alba* EO against the radical elongation of *B. napus*, *H. vulgaris*, *L. multiflorum* and *T. pratense.* The results are the mean of three experiments ± SD. * *p* < 0.05; ** *p* < 0.01; *** *p* < 0.001; **** *p* < 0.00001 compared with the control (ANOVA followed by Dunnet’s multiple comparison test).

Figure 1 and Figure 2 report the herbicidal properties of the EO on the germination and radical elongation of the weeds, *Lolium multiflorum* and *Trifolium pratense*, and on the crops *Brassica napus* and *Hordeum vulgare*. The EO demonstrated variable phytotoxic effects in a dose-dependent manner. The EO was active on all the seeds tested both on the germination and on the radical elongation. However, the effect was slightly higher for the second event. At the highest concentrations (1000 and 500 μg/mL), the EO caused total inhibition (100%) of the germination of *B. napus* and *H. vulgare*. A 100% inhibition also occurs for *T. pratense* at the concentration of 1000 μg/mL but not for the second higher concentration (48.20% of inhibition). For *L. multiflorum*, there are high inhibitions but not at 100%: 76.29% at 1000 μg/mL and 58.77% at 500 μg/mL. At the lowest concentrations (250 and 125 μg/mL), the effect was reduced: the highest inhibition values were found for *B. napus* (89.29 and 87.50%, respectively).

The EO causes 100% inhibition of the radical elongation of *B. napus* at all concentrations, while at the highest concentrations (1000 and 500 μg/mL), it showed a total inhibition for *H. vulgaris* and a total inhibition for *T. pratense* only for the highest concentration. At the lowest concentrations, the activity significantly decreases. The highest inhibitions at 250 and 125 μg/mL were found for *B. napus* (100% for both concentrations), followed by *H. vulgare* (74.47 and 27.66%, respectively) and *L. multiflorum* (51.42 and 25.71%, respectively). Instead, on *T. pratense*, the inhibition was 6.67% for the concentration 250 μg/mL and 0% at 125 μg/mL.

### 2.3. Ecotoxicity

The mortality of the model organisms after exposure to EO from *A. herba*-*alba* was assessed using the same concentrations as those used in the phytotoxicity test (1000 μg/mL, 500 μg/mL, 250 μg/mL and 125 μg/mL). The mortality index for the semi-aquatic organism *Tubifex tubifex* was 0% at all applied EO concentrations, even after prolonged exposure (assessments performed from 3 min up to 24 h). Furthermore, for the soil representative *Eisenia foetida*, no mortality was observed at any of the used concentrations after 48 h of exposure. Aquatic larvae of non-biting mosquitoes of *Chaoborus* spp. showed, after 48 h, an average mortality of 29% after exposure to the highest EO concentration used in the phytotoxicity tests. The second highest concentration caused a mortality of 8%. The lowest concentrations did not cause mortality for these aquatic organisms. All results were compared with a control test performed using distilled water, in which the survival of the organisms was 100%.

## 3. Discussion

Several papers are available concerning the chemical composition of EO from *A. herba*-*alba*, whose results are in partial agreement with what is highlighted in this work. Younsi and collaborators [42] studied the composition of the EOs from 80 individuals from 8 populations growing in different geographical areas of Tunisia. Four chemotypes (*trans*-sabinyl acetate, α-thujone/*trans*-sabinyl acetate, camphor and α-thujone/camphor/β-thujone) were identified, and oxygenated monoterpenes were the predominant class (73.6–89.7%). The composition is similar to that reported in this work, in which the EO belongs to the camphor chemotype, with the predominance of oxygenated monoterpenes and the presence of many of the main components highlighted in the work of Younsi, although in different amounts. An EO obtained by the SPME technique [43] was found to be rich in oxygenated monoterpenes (64.9%, slightly lower than the 79.35% present in this work), and among the main compounds, it presented α-thujone (37.9%), germacrene D (16.5%), 1,8-cineole (8.4%), β-thujone (7.8%) and bicyclogermacrene (5.8%). Some of these are also present in the EO studied in this work: α-thujone but in very different quantities (37.9 vs. 9.60%) and β-thujone and eucalyptol in similar quantities (7.8 vs. 8.38 and 8.4 vs. 8.02%, respectively). The same authors analyzed EOs from leaves, stems and roots of a Tunisian *A. herba*-*alba* [44]. All EOs present a considerable presence of α-thujone (18.2–45.5%), β-thujone (5.7–11.4%), camphor (6.8–22.9%) and 1,8-cineole (1.7–8.3%). These compounds agree with the main compounds of the EO of this work, in very similar amounts (except α-thujone, in lower amounts). In 2015, a comparative study was carried out on the composition of the EOs of *A. herba*-*alba* from different locations [45]. In all the EOs, oxygenated monoterpenes were the main class (65.5–70.3%), as in this work. The main components were camphor (0.64–31.51%), fenchol (7.51–13.85%), germacrene D (2.24–5.89%), limonene (4.57–7.29%), nordavanone (1.26–9.44%), α-thujone (11.62–13.93%) and β-thujone (0.16–8.04%). Camphor, α-thujone and β-thujone are also the main components in the EO studied in this work, with similar quantities except for α-thujone (present in smaller quantities). Finally, there is a contribution regarding the EO composition of *A. herba*-*alba* from Spain [46], which reports as the main components camphor (15.0%) and 1,8-cineole (13.3%).

Few contributions are available regarding the possible herbicidal activity of the EO of *A. herba*-*alba*. The allelopathic potential of an EO from an Algerian *A. herba*-*alba* on nine weeds and two wheat varieties was tested. The results showed activity on all the seeds considered, but it varied both according to the seed and the concentrations used. The highest inhibitory activity, both on germination and radical elongation, was observed on *Silybum marianum* (L.) Gaertn., *Scorzonera laciniata* Jacq., *Mantisalca salmantica* (L.) Briw. & Cavill., *Bromus madritensis* L., *Ammi visnaga* (L.) Lam. and *Diplotaxis tenuifolia* (L.) DC., while it was lower on *Hordeum murinum* L. and *Sinapis arvensis* L. However, it had a stimulating effect on *Avena fatua* L. [6]. A strong inhibitory activity on the growth of crops such as *Lactuca sativa* L. and *Raphanus sativus* L was instead highlighted for an EO from the leaves of *A. herba*-*alba*, able to completely inhibit the germination of these two species [43]. Finally, the phytotoxic properties of an EO of *A. herba*-*alba* on *Raphanus sativus*, *Lepidium sativum* L., *Sinapis arvensis*, *Triticum durum* L. and *Phalaris canariensis* L. seeds were reported [47]. The concentrations used were lower than those used in this work and the EO was inactive in inhibiting germination but more active in inhibiting radical elongation. At the highest doses, a stimulating effect on *R. sativus* was noted. These results agree with the greater activity of the EO regarding radical elongation rather than germination and show how effectively the EO has effects that manifest themselves on many weeds but also on some crops. EOs are recognized as effective phytotoxic agents [48] and act in this sense through mechanisms involving different communication and interaction signals between plants that, through phenotypic modifications, see their germination and root elongation processes influenced [49]. The activity is obviously linked to the composition: there are many volatile compounds active in this sense, and among these, some of the main components of the EOs studied in this article can contribute to phytotoxicity. In fact, camphor [28], α- and β-thujone and 1,8-cineole have been reported for their phytotoxic activity [48,49,50]. There are no studies in the literature analyzing the ecotoxic potential of the EO of *A. herba*-*alba* using the organisms of this work. Although it is not excluded that *A. herba*-*alba* EO can pose some toxicity to non-target organisms, concentrations which impacted the germination or root growth of model plants within the phytotoxic bioassay were not toxic to soil nor to semiaquatic representatives at all. There was a slight mortality for aquatic organisms, which highlights the need for further studies and, in general, for caution in the practical application of EOs and adherence to correct application practices, just as with conventional herbicides [7,8,9]. The ecotoxicity effects manifested, although minimal, are linked to the composition and, in particular, to camphor [51,52,53]. Specific information on the ecotoxicity of thujone is not present in the literature. However, the works on this subject used different techniques and organisms, and therefore, a direct comparison with the present work is not yet possible.

## 4. Materials and Methods

### 4.1. Plant Materials

*Artemisia herba*-*alba* was collected at the beginning of September 2023 (flowering stage) in the city of Siliana (Bargou region, latitude 36°4′55″ N longitude 9°22′29″ E), North West of Tunisia. The plant was identified by Dr. I. Amri, and a voucher specimen is stored in the herbarium section of the Institut National de la Recherche en Génie Rural, Eau et Forest, INRGREF, Tunis. The plant was air-dried for 15 days and stored in a dark, humidity-controlled environment until it was ready for use.

### 4.2. Extraction of the EO

Aerial parts were steam-distilled for 2 h, according to the method reported by the European Pharmacopoeia [54]. The EO was solubilized in *n*-hexane, filtered over anhydrous sodium sulphate and stored under N_2_ at +4 °C in the dark until tested and analyzed. The EO yield was 0.1% on dry weight.

### 4.3. GC/MS Analyses

GC-MS analysis was performed using an Agilent 6850 Ser. II Apparatus (Santa Clara, CA, USA) equipped with a DB-5 fused silica capillary column (30 m× 0.25 mm; 0.25 μm film thickness) and connected to an Agilent Mass Selective Detector (MSD 5973) (Santa Clara, CA, USA) with an ionization voltage of 70 V and an ion multiplier energy of 2000 V. The mass spectra were scanned in the range of 40–500 amu, with five scans per second. The chromatographic conditions were as reported above, and the transfer line temperature was 295 °C. The analysis was conducted on a scheduled basis: 5 min isothermally at 40 °C; subsequently, the temperature was increased by 2 °C/min until 270 °C, and finally, it was kept in isotherm for 20 min. The analysis was also performed on an HP Innowax column (50 m × 0.20 nm; 0.25 μm film thickness). In both cases, He was used as a carrier gas (1.0 mL/min). Most of the components were identified by comparing their Kovats indices (Ki) with those of the literature [55,56,57,58] and by a careful analysis of the mass spectra compared to those of the pure compounds available in our laboratory or to those present in the NIST 02 and Wiley 257 mass libraries [59]. The Kovats indices were determined in relation to a homologous series of n-alkanes (C10–C35), under the same operating conditions. For some compounds, the identification was confirmed by co-injection with standard samples. Components’ relative concentrations were calculated by peak area normalization. Response factors were not considered.

### 4.4. Phytotoxic Activity

The phytotoxic activity was evaluated against the seed germination and radicle emergence/elongation of two weeds, *Lolium multiflorum* Lam. and *Trifolium pratense* L., and two horticultural crops, *Brassica napus* L. and *Hordeum vulgare* L. The seeds were purchased from Blumen group srl (Emilia-Romagna, Italy). The seeds were surface-sterilized in 96% ethanol for 15 s and sown in Petri dishes (Ø = 90 mm) on three layers of Whatman filter paper. They were impregnated with 7 mL of deionized water, and then 7 mL of a water–acetone mixture (99.5:0.5, *v*/*v*) was used as the control since EOs were dissolved in this mixture due to their lipophilicity. Finally, 7 mL of the EO solution at different concentrations (1000, 500, 250 and 125 μg/mL) was tested. The controls carried out with the water–acetone mixture alone showed no differences in comparison to the controls in water alone. The germination conditions were 20 ± 1 °C, with a natural photoperiod. Seed germination was checked in Petri dishes every 24 h. A seed was considered germinated when the protrusion of the root became evident [60]. On the fifth day (after 120 h) for *B. napus* and *H. vulgare* and on the seventh day (after 168 h) for the other seeds, the effects on radicle elongation were determined by measuring the root length in cm. Each evaluation was replicated three times, using Petri dishes containing 10 seeds each. The data were expressed as the mean ± standard deviation for both germination and radicle elongation.

### 4.5. Ecotoxicity

To test the ecotoxicity of EO, three model organisms, representing aquatic, semi-aquatic and soil organisms, were exposed to the same EO concentrations as applied in the phytotoxicity bioassay. Water was used as a control (0% mortality). Aquatic glassworms—larvae of the non-biting mosquito of *Chaoborus* spp. (Diptera: Chaoboridae)—are harmless representatives of the water invertebrates. They were subjected to the laboratory testing of mosquito larvicides following the standard methodology suggested by the World Health Organization [61], with a slight modification [62]. Mortality was evaluated after 24 h. For each EO concentration, 10 repetitions with 10 individuals were performed (n = 100).

Semiaquatic sludge worm *Tubifex tubifex* Müller, 1774 (Annelida, Oligochaeta: Tubificidae)—a standard model organism that is widely used in ecotoxicological studies—was tested for acute toxicity using an express 3 min test following Tichý et al. [63]. For each EO concentration, 10 repetitions with 10 individuals were performed (n = 100).

As the zero mortality was observed after 3 min, prolonged exposition was applied and the number of death worms was checked after 10, 20, 30, 60, 180 and 240 min and then after 24 h.

The earthworm *Eisenia foetida* Savigny, 1826 (Annelida, Oligochaeta: Lumbricidae) was used as a representative of soil organisms. Ecotoxicity was tested using the contact method according to OECD [64]: the individuals were placed in a Petri dish on filter paper soaked with 2 mL of EO dilution, and mortality was evaluated after 48 h. For each EO concentration, 10 repetitions were performed (n = 10 individuals).

### 4.6. Statistical Analysis

Statistical analysis was performed by analysis of variance (ANOVA) using GraphPad Prism 6.0 (Software Inc., San Diego, CA, USA), expressed as the mean ± standard deviation (S.D.). The results were compared to the untreated control and considered statistically significant, by Dunnett’s test, when *p* < 0.05 (* *p* < 0.05, ** *p* < 0.01, *** *p* < 0.001, **** *p* < 0.0001).

## 5. Conclusions

In this study, data were collected regarding the composition, phytotoxicity and impact on environmental safety of the EO of *A. herba*-*alba* from Tunisia. The results demonstrate strong phytotoxicity activity both for what concerns the inhibitory action on germination and root elongation, especially for *T. pratense*. Furthermore, preliminary tests on a possible environmental toxicity gave encouraging results, since the EO did not demonstrate any danger for the reference organisms used. The results lay the foundation for considering the EO of *A. herba*-*alba* as a potential weed growth control agent that, at the same time, appears to have a less dangerous environmental impact than other chemical control agents.

## Figures and Tables

**Table 1:** Chemical composition of the EO.

		%	Retention Time (min)	Ki ^a^	Ki ^b^	Identification ^c^
1	Santolina triene	0.18	11.116	836	1036	1, 2
2	Tricyclene	0.32	11.638	843	1012	1, 2
3	α-Pinene	0.99	12.472	854	1025	1, 2, 3
4	Camphene	4.95	13.456	867	1068	1, 2, 3
5	Sabinene	0.74	15.199	874	1122	1, 2
6	Dehydro-1,8-cineole	0.06	16.401	905	1192	1, 2
7	Mesitylene	0.28	16.709	909	1251	1, 2
8	α-Phellandrene	0.06	17.341	917	1168	1, 2, 3
9	Yomogi alcohol	0.20	17.616	920	1395	1, 2
10	α-Terpinene	0.13	18.241	929	1178	1, 2, 3
11	1,2,4-Trimethyl benzene	0.17	18.605	933		1, 2
12	o-Cymene	0.87	18.954	938	1310	1, 2
13	1,8-cineole	8.02	19.311	942	1211	1, 2, 3
14	Santolina alcohol	0.25	19.989	951		1, 2
15	γ-Terpinene	0.15	21.344	969	1245	1, 2, 3
16	Terpinolene	1.11	21.527	971	1282	1, 2, 3
17	*trans*-Sabinene hydrate	0.29	22.165	979	2092	1, 2
18	α-Thujone	9.60	24.74	1010	1423	1, 2, 3
19	Santolina epoxide	0.18	25.002	1013		1, 2
20	β-Thujone	8.38	25.713	1023	1440	1, 2, 3
21	Chrysantenone	0.81	26.078	1027	1508	1, 2
22	*trans*-*p*-Menth-2-en-1-ol	0.72	26.404	1032	1584	1, 2
23	*cis*-Verbenol	0.07	26.812	1037	1660	1, 2
24	Camphor	26.02	27.518	1047	1515	1, 2, 3
25	Camphene hydrate	0.21	27.902	1052	1602	1, 2
26	β-Pinene oxide	0.11	28.454	1059	1364	1, 2
27	Pinocarvone	1.46	28.752	1063	1576	1, 2
28	Borneol	3.60	29.14	1069	1670	1, 2, 3
29	*p*-Mentha-1,5-dien-8-ol	0.18	29.47	1073	1674	1, 2
30	Terpinen-4-ol	0.53	29.861	1078	1601	1, 2, 3
31	α-Thujenal	0.09	30.278	1084		1, 2
32	(*E*)-Isocitral	0.32	30.396	1085		1, 2
33	Myrtenal	1.00	30.846	1091	1632	1, 2
34	*cis*-Piperitol	0.13	31.13	1095	1750	1, 2
35	Myrtenol	0.25	31.265	1097	1790	1, 2
36	Verbenone	0.76	31.942	1100	1726	1, 2
37	*trans*-Piperitol	0.27	32.079	1102	1710	1, 2
38	*trans*-Carveol	0.10	32.303	1105	1720	1, 2
39	*cis*-Carveol	0.06	32.98	1115	1854	1, 2
40	(*E*)-Ocimenone	0.17	33.159	1117		1, 2
41	Car-3-en-2-one	1.49	34.405	1135		1, 2
42	Piperitone	0.48	35.181	1146	1730	1, 2
43	*cis*-Verbenyl acetate	0.83	35.692	1153		1, 2
44	*p*-Mentha-1,8-dien-3-one	1.62	36.41	1164		1, 2
45	Bornyl acetate	0.79	37.241	1176	1579	1, 2
46	*trans*-Pinocarvyl acetate	0.10	37.865	1184	1661	1, 2
47	*cis*-Pinocarvil acetate	0.28	38.262	1190		1, 2
48	(*Z*)-Patchenol	0.62	38.573	1195		1, 2
49	(*E*)-Patchenol	2.80	39.504	1203		1, 2
50	δ-Elemene	0.32	40.493	1218	1469	1, 2
51	*cis-*Carvyl acetate	0.70	41.82	1238		1, 2
52	α-Copaene	0.18	42.891	1254	1491	1, 2
53	Piperitenone	5.29	44.78	1282	1909	1, 2
54	Daucene	0.59	45.562	1294	1495	1, 2
55	β-Cubebene	0.25	46.187	1297	1542	1, 2
56	β-Longipinene	0.10	47.113	1312		1, 2
57	β-Cedrene	0.12	47.624	1321		1, 2
58	Aromadendrene	0.12	48.052	1328	1620	1, 2
59	*allo*-Aromadendrene	0.24	49.139	1345	1660	1, 2
60	Germacrene D	0.38	49.337	1348	1708	1, 2
61	Chrysantenyl 2-methylbutanoate	0.38	49.871	1357		1, 2
62	δ-Guaiene	0.35	50.267	1363		1, 2
63	Bornyl 2-methylbutanoate	0.07	50.986	1375	1241	1, 2
64	δ-Amorphene	0.19	52.007	1391		1, 2
65	5-Isopropenyl-2-methyl-2-cyclohexen-1-yl pivalate	0.06	52.784	1399		1, 2
66	Palustrol	0.28	54.352	1426	1930	1, 2
67	Spathulenol	1.24	55.062	1438	2127	1, 2
68	Caryophyllene oxide	0.70	55.211	1446	1986	1, 2
69	Viridiflorol	0.82	55.801	1450	2090	1, 2
70	*cis*-Carvyl angelate	0.21	56.04	1454		1, 2
71	*cis*-Eudesm-6-en-11-ol	0.24	56.403	1460		1, 2
72	Junenol	0.07	57.18	1474		1, 2
73	γ-Eudesmol	0.33	57.896	1486	2176	1, 2
74	Ylangenal	0.23	58.196	1491		1, 2
75	Caryophylla-4(12),8(13)-dien-5α-ol	0.12	58.319	1493	2324	1, 2
76	Isospathulenol	0.15	58.505	1496	2228	1, 2
77	τ-Cadinol	0.22	58.746	1493	2151	1, 2
78	α-Eudesmol	0.34	59.026	1498		1, 2
79	α-Cadinol	0.61	59.408	1505	2224	1, 2
80	(1*R*,7*S*)-Germacra-4(15),5,10(14)-trien-1β-ol	0.18	61.115	1536		1, 2
81	Icosa-9,11-diyne	0.10	72.287	1735		1, 2
Total		97.98				
Monoterpene hydrocarbons		9.95				
Oxygenated monoterpenes		79.35				
Sesquiterpene hydrocarbons		2.84				
Oxygenated sesquiterpenoids		5.74				
Others		0.10				

^a,b^ Kovats retention indices determined relative to a series of n-alkanes (C10–C35) on the apolar HP-5 MS and the polar HP Innowax capillary columns, respectively. ^c^ Identification method: 1 = comparison of the Kovats retention indices with published data, 2 = comparison of mass spectra with those listed in the NIST 02 and Wiley 275 libraries and with published data; 3 = co-injection with authentic compounds.

## Data Availability

The original contributions presented in the study are included in the article. Further inquiries can be directed to the corresponding author.
